# 

**Published:** 1914-09-19

**Authors:** 


### THE NEW REGIUS PROFESSOR AT ABERDEEN.

On the recommendation of the Secretary for Scotland the King has appointed Theodore Shennan, M.D., F.R.C.S.E., pathologist to the Royal Infirmary of Edinburgh, Regius Professor of Pathology in the University of Aberdeen. Professor Shennan, who is only forty-five years of age, graduated in medicine and surgery at Edinburgh in 1890, and since 1895 has given up his time to pathology, which he has studied in London, America, Munich, Copenhagen, and other centres at home and abroad.

### THE RELIEF FUND AND COUNTY CRICKET.

Several of the most famous county cricketers, including Dr. W. G. Grace, Messrs. P. F. Warner, T. Hayward, and G. Hirst, have made the following suggestion, on behalf of the National Relief Fund, to the cricket-loving public :—

"We have before us as we write the vision of many a fair English cricket ground packed with eager multitudes. . . . It is this thought which has given rise to this particular appeal. We ask all those who have watched us play, and who have cheerfully paid their half-crowns, shillings, and sixpences as gate-money, to step forward and contribute over again their half-crowns, shillings, and sixpences to the Prince's Fund, out of gratitude for the enjoyment the cricket field has given them in the past. Let everyone who has followed cricket recall to mind the matches he has witnessed and enjoyed, and let each one contribute according to the pleasantness of his memories. Then we shall have for those whom the war has robbed not only of happiness, but even of the means of livelihood, a truly royal sum."

### Oldham Royal Infirmary.

THE above institution is entitled to receive £5,000, free of legacy duty, from the late Mr. H. L. Hargraves, a memoir of whom appeared in our issue of August 29 (p. 606), for the Endowment Fund, under his will. After declaring full discretionary powers to his trustees and the satisfaction of various bequests, the rest and residue of his estate is to be held in trust for the Oldham Royal Infirmary, with a view, after consultation, to provide a convalescent home. Should there be no agreement, then it is to be held in suspense, and the surplus of income to accumulate at compound interest with the resulting income, for the benefit of the infirmary.

### The Book Market.

LIST OF NEW BOOKS RECEIVED FROM THE FOLLOWING PUBLISHERS:—

Humphrey Milford, for the Harvard University Press: "The Care and Feeding of Children." By J. Lovett Morse, M.D. 2s. 6d. net.

The Scientific Press, Ltd.: "Notes on Skin Diseases." By David Walsh, M.D. 1s. net. "The Tuberculosis Handbook." By A. H. G. Burton, M.D. 2s. 6d. net. "Practical Medical Electricity." By Alfred C. Norman, M.D. 5s. net. "Standard Prescriptions for Insurance Practice." Compiled by C. H. Gunson, M.B., Ch.B. 1s. net.

W. B. Saunders and Co.: "Anoci-association." By G. W. Crile, M.D., and W. B. Lower, M.D. 13s. net.

Thacker, Spink and Co., Calcutta: "Tropical Hygiene." Second Edition. By the Hon. Surg.-Gen. Sir Pardey Lukes, K.C.S.I., and Major R. J. Blackham, C.I.E., R.A.M.C.

John Wright and Sons, Ltd., Bristol: "Manual for Women's Voluntary Aid Detachments." Third Edition. By P. C. Gabbett, M.R.C.S. 1s. net.

George Allen and Unwin, Ltd.: "A Text-book of Insanity and other Mental Diseases." Second Edition. By C. A. Mercier, M.D. 7s. 6d. net.

John Murray: "Nature and Nurture in Mental Development. By F. W. Mott, M.D., F.R.S. 3s. 6d. net. "Practical Tropical Sanitation." By W. A. Muirhead, R.A.M.C. 10s. 6d. net.

Rates of Subscription: Twelve months, 6/6; Six months, 4/-; Three months, 2/-, post free to any address in the United Kingdom. Abroad, Twelve months, 8/8; Six months, 5/-; Three months, 2/6, post free.—Address The Subscription Dept., "The Hospital," The Hospital Building, 28/29 Southampton Street, Strand, London W.C.

